# *Ophiocordyceps salganeicola*, a parasite of social cockroaches in Japan and insights into the evolution of other closely-related *Blattodea*-associated lineages

**DOI:** 10.1186/s43008-020-00053-9

**Published:** 2021-02-05

**Authors:** João P. M. Araújo, Mitsuru G. Moriguchi, Shigeru Uchiyama, Noriko Kinjo, Yu Matsuura

**Affiliations:** 1 Tropical Biosphere Research Center, University of the Ryukyus, Nishihara, Okinawa Japan; 2 School of Forest Resources and Conservation, University of Florida, Gainesville, Florida USA; 3 Okinawa University, Naha, Okinawa Japan; 4 The Japanese Society of Cordyceps Research, Tokyo, Japan

**Keywords:** *Ascomycota*, *Hypocreales*, Cockroaches, Termites, Entomopathogenic fungi, Host-jumps, *Ophiocordyceps sinensis*

## Abstract

The entomopathogenic genus *Ophiocordyceps* includes a highly diverse group of fungal species, predominantly parasitizing insects in the orders *Coleoptera, Hemiptera, Hymenoptera* and *Lepidoptera*. However, other insect orders are also parasitized by these fungi, for example the *Blattodea* (termites and cockroaches). Despite their ubiquity in nearly all environments insects occur, blattodeans are rarely found infected by filamentous fungi and thus, their ecology and evolutionary history remain obscure. In this study, we propose a new species of *Ophiocordyceps* infecting the social cockroaches *Salganea esakii* and *S. taiwanensis*, based on 16 years of collections and field observations in Japan, especially in the Ryukyu Archipelago. We found a high degree of genetic similarity between specimens from different islands, infecting these two *Salganea* species and that this relationship is ancient, likely not originating from a recent host jump. Furthermore, we found that *Ophiocordyceps* lineages infecting cockroaches evolved around the same time, at least twice, one from beetles and the other from termites. We have also investigated the evolutionary relationships between *Ophiocordyceps* and termites and present the phylogenetic placement of *O.* cf. *blattae*. Our analyses also show that *O. sinensis* could have originated from an ancestor infecting termite, instead of beetle larvae as previously proposed.

## INTRODUCTION

The genus *Ophiocordyceps* (*Hypocreales*, *Ophiocordycipitaceae*) comprises species typically pathogenic to insect hosts. Recently, however, there are reports of beneficial, endosymbiotic species of sap-sucking hemipterans hosts (Quandt et al. 2014; Gomez-Polo et al. 2017; Matsuura et al. 2018). The genus was erected by Petch (1931) to accommodate species of *Cordyceps* exhibiting clavate asci containing spores that do not disarticulate into partspores, contrasting with “the majority of the species of Cordyceps which have been described” at that time, exhibiting cylindrical asci and spores that readily disarticulate into numerous short partspores upon maturity. The diversity of *Ophiocordyceps* has been increasingly unraveled in the last decade, especially with discoveries of species associated with *Hymenoptera*, *Lepidoptera* and *Hemiptera* (Araújo et al. 2016, 2018; Luangsa-ard et al. 2018). However, our knowledge about species associated with blattodean insects (cockroaches and termites) is still restricted, especially regarding cockroach parasites. Currently, we know of only 11 species infecting termites, i.e. *O. bispora* (Stifler 1941); *O. octospora* (Blackwell and Gilbertson 1981); *O. communis* (Sung et al. 2007); *O. asiatica*, *O. brunneirubra*, *O. khokpasiensis*, *O. koningsbergeri*, *O. mosingtoensis*, *O. pseudocommunis*, *O. pseudorhizoidea,* and *O. termiticola* (Tasanathai et al. 2019). Furthermore, there are only three species described infecting cockroaches, i.e. *O. blattae* from Sri Lanka (formerly Ceylon) – (Petch 1931); *O. blattarioides* from Colombia – (Sanjuan et al. 2015) and *O. salganeicola* sp. nov. from Japan – this study).

The cockroaches (*Blaberidae, Blattodea*) are ubiquitous organisms occupying almost all habitats where insects occur (Schall et al. 1984). They play a key ecological role as decomposers, with many examples of species living within and feeding on rotting wood, including a lineage of social species that evolved into the termites (Bell et al. 2007; Maekawa et al. 2008; Bourguignon et al. 2018; Evangelista et al. 2019). Parasitism by microsporidians and protozoans on these insects is relatively common and known for more than a century (Crawley 1905; Woolever 1966; Purrini et al. 1988). However, the cases of infection by filamentous fungi on cockroaches have been rarely reported.

**Fig. 1:**
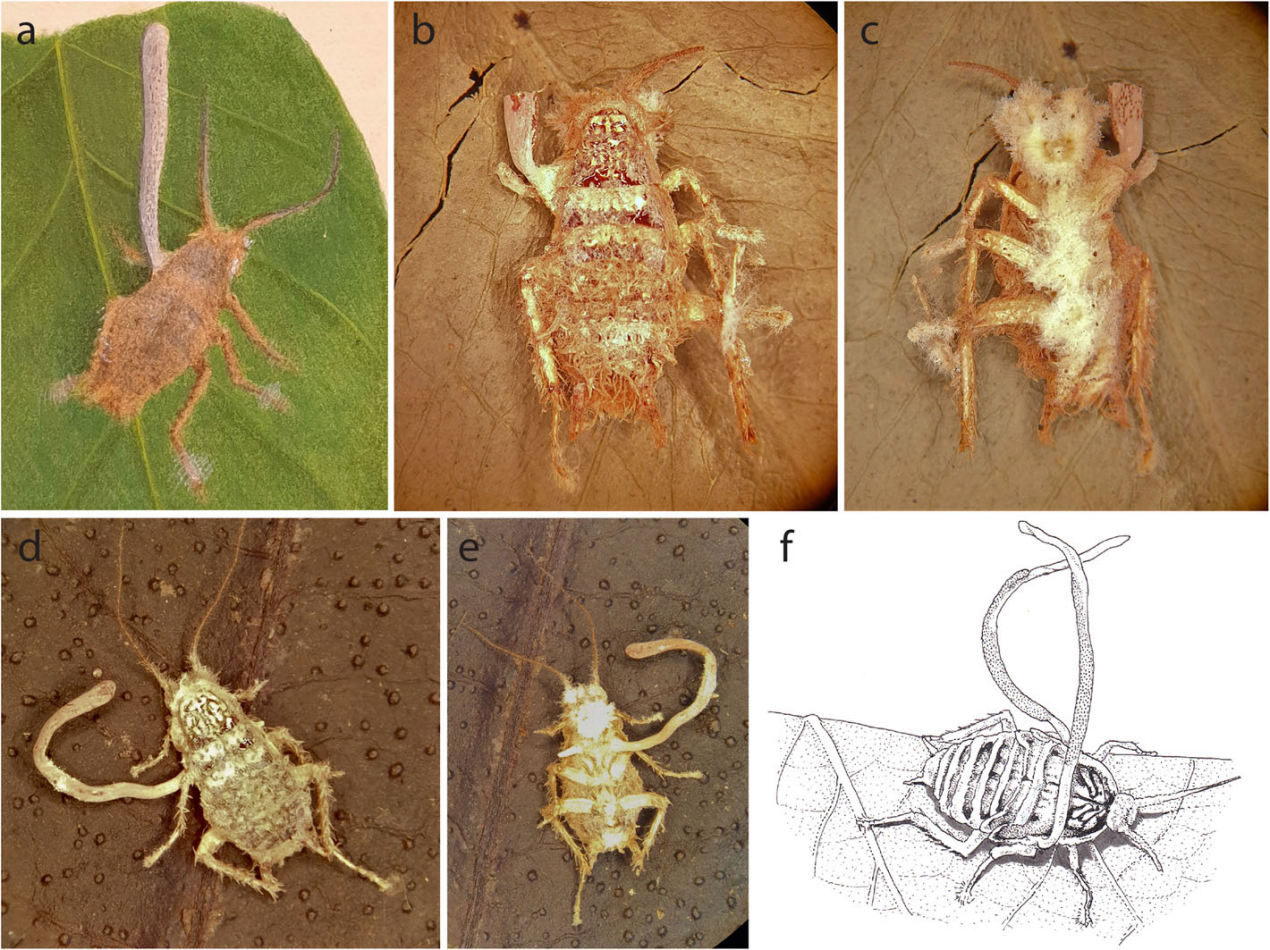
*Ophiocordyceps blattae* and *O.* cf. *blattae*. **a** the original illustration of *O. blattae* showing the ascoma emerging laterally from the host thorax (Petch 1924). **b** and **c** (K98612 – holotype deposited in the Kew Gardens Fungarium). **d** and **e** Additional specimen collected by Petch in 1914, same location as the holotype K(M)264510. **f** *O.* cf. *blattae* from Thailand used in this study with two ascomata arising laterally on both sides (deposited at Biotech Fungal Collections as MY34765) (del. M. G. Moriguchi). **a**–**e**; images by Lee Davis, Royal Botanic Garden, Kew

Although examples of *Ophiocordyceps* parasitizing *Blattodea* are scarce, the type of the genus is *O. blattae* infecting a cockroach identified as *Blatta germanica* (Fig. 1, adapted from Petch 1924). Only two specimens were originally collected in Sri Lanka (See Fig. 1a-d) with recent few records from Thailand (Luangsa-ard et al. 2018). Another example of an *Ophiocordyceps* infecting a cockroach is *O. blattarioides* (syn. *Paraisaria blattarioides*; Mongkolsamrit et al. 2019). This species was described from Colombia with records in Belize and tropical lowlands in the eastern (Amazonian) Ecuador (Sanjuan et al. 2015). Both *O. blattae* and *O. blattarioides* exhibit striking morphological and ecological differences. For example, *O. blattae* forms a cylindric ascoma producing elongated-fusoid ascospores, which do not disarticulate into part-spores, measuring 50–80 × 3–4 μm, while *O. blattarioides* has a stalk bearing a globoid fertile part at the tip, producing spores that disarticulate into part-spores of 6–12 × 1.5 μm. The host death location is also distinct with *O. blattae* occuring on the underside of leaves while *O. blattarioides* is found buried in the leaf litter. Besides these studies, there is no detailed information on the evolution and ecology of cockroach-associated entomoparasitic fungi.

In this study, we propose a new species of *Ophiocordyceps* that parasitizes two social wood-feeding cockroach species distributed in the southwestern part and Nansei Islands of Japan, both living inside decaying logs. We provide morphological, molecular and ecological data to support the new species proposal with insights into the evolutionary origins of the closely related parasitic fungi of cockroaches and termites. We also present, for the first time, the phylogenetic position of *O.* cf. *blattae*.

## MATERIALS AND METHODS

### Sampling

**Fig. 2:**
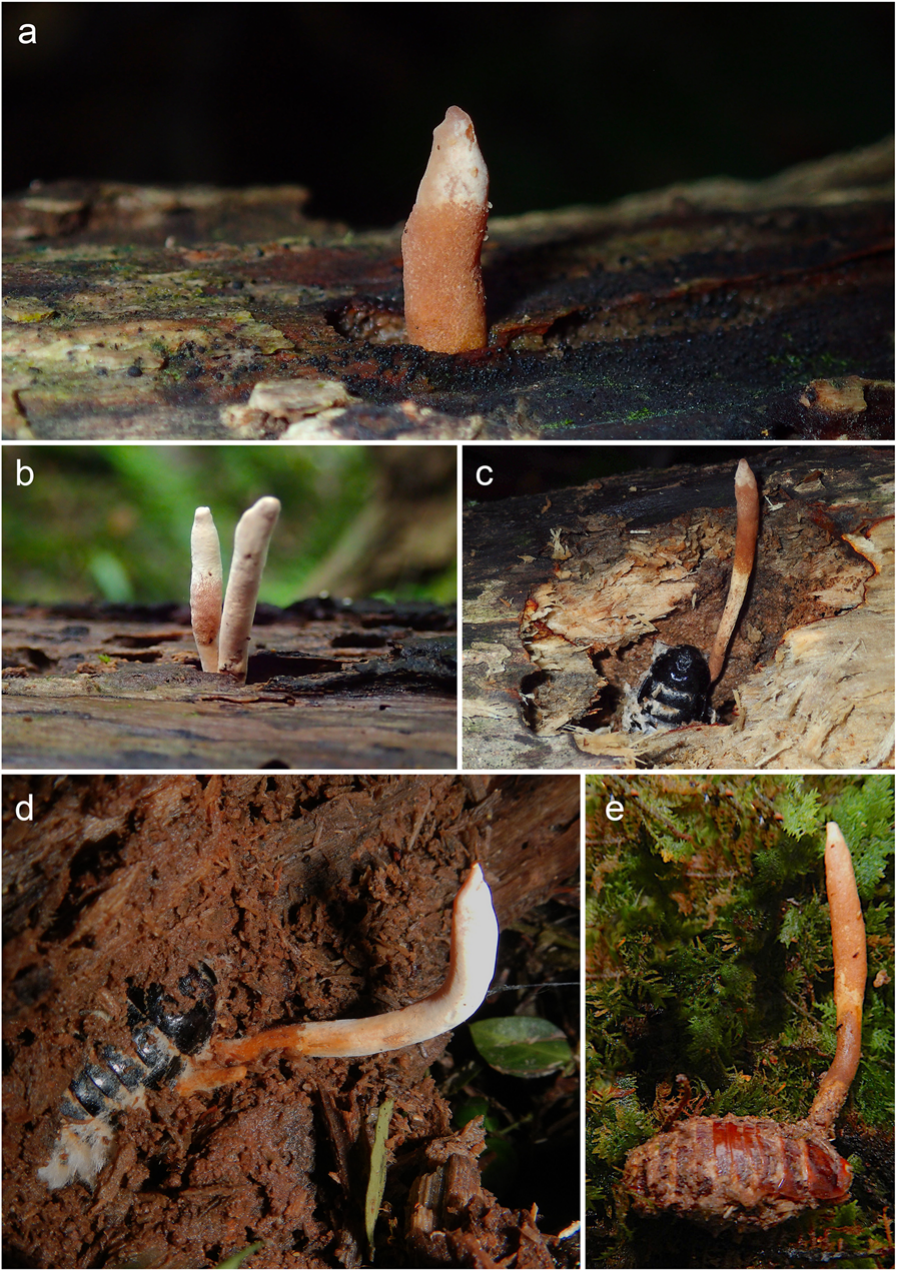
*Ophiocordyceps salganeicola* emerging from *Salganea esakii* in its natural habitat in Yakushima island, Kagoshima. **a** and **b** Visible part of the ascoma, emerging from a hole in the wood. **c** and **d** Hosts buried in the wood, visible only after digging a few centimeters. **e** Infected host in its very rare nymphal stage

Surveys were undertaken in the Japanese warm temperate and subtropical evergreen forests mainly consisting of trees belonging to *Fagaceae*, *Lauraceae* and *Theaceae* in Kunigami-son, Okinawa, Yakushima, Kagoshima and Nobeoka, Miyazaki. The parasitized cockroach samples of two host species, namely *Salganea esakii* and *S. taiwanensis,* were mainly collected in the small humid valley or riparian forests where *Castanopsis sieboldii*, *Distylium racemosum* and *Schefflera heptaphylla* grow, but also in the secondary forest harboring *Alnus japonica* after the deforestation of *Castanopsis* in Okinawa. The specimens used in this study were always found hidden inside soft rotten logs or large fallen branches of those trees, with only the fungus emerging (Fig. 2). The infected cockroaches, and the substrata they were attached to, were collected in plastic containers and transported to the laboratory. Some specimens were investigated immediately after the collection, while others were dried and preserved for many years before being analyzed. The specimens were photographed individually, using a Canon 7D camera, equipped with an EF-100 mm macro lens or a MP-E 65 mm (5X) lens with a MT-24EX Canon macro lite flash attached.

### Collections

**Fig. 3:**
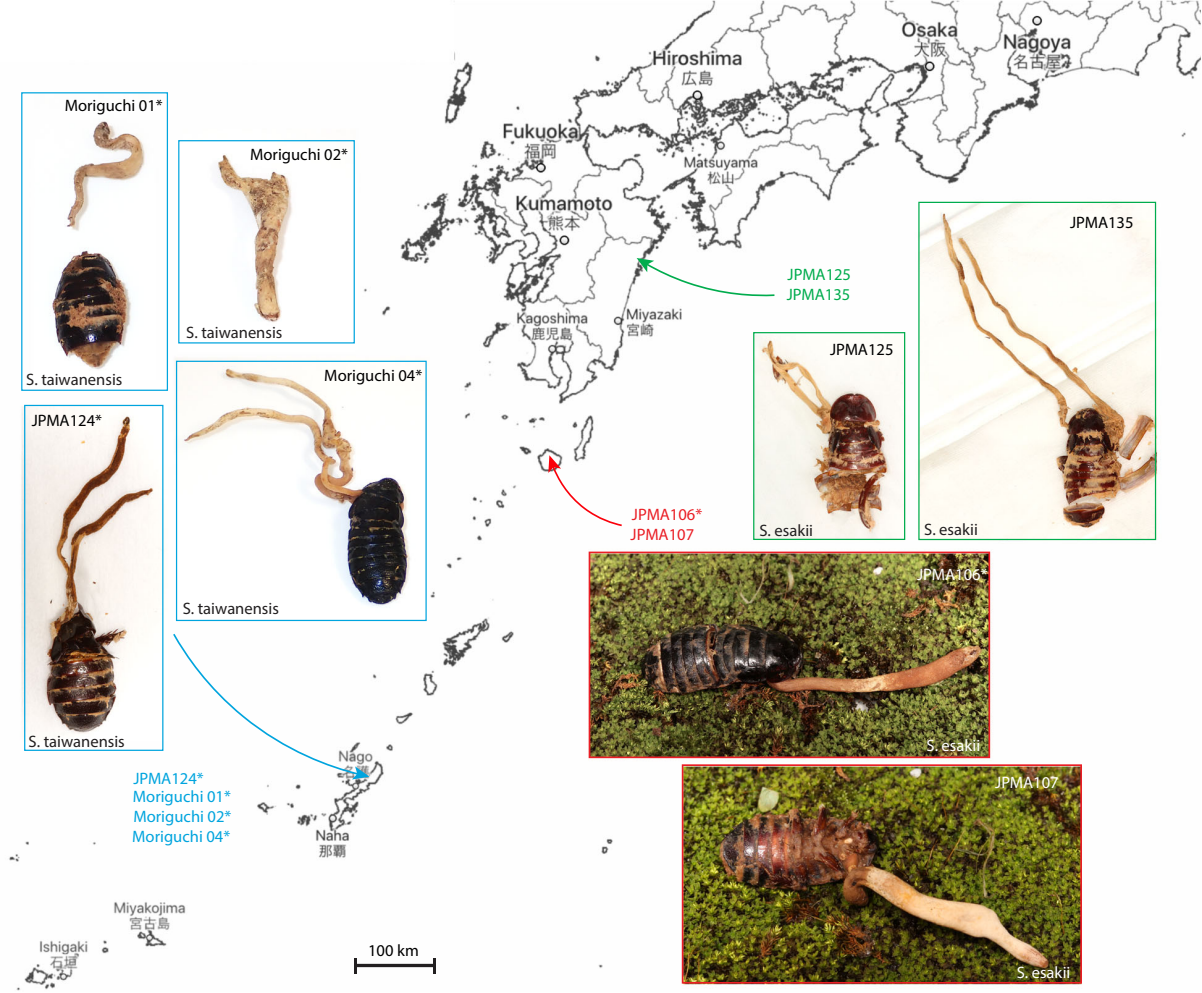
Southwestern part of Japan showing collection sites of *O. salganeicola* specimens used in this study. Specimen details are indicated in Table 1. The map was retrieved and edited from Geospatial Information Authority of Japan

We collected 26 samples in two locations in Okinawa, 27 in Yakushima island, Kagoshima, and five in Miyazaki. Four specimens from Yonahadake and Kunigami (Okinawa) and two from Kagoshima were used for DNA extraction and sequencing for each prefecture (see Fig. 3, Table 1). Almost all fungal specimens were collected from adults of *S. esakii* and *S. taiwanensis* between April – June from 2004 to 2019.

**Table 1:** Host association, collection date, collection site and GenBank accession numbers for the specimens used in this study

ID No.	Host insect (fungal morph/stage)	Collection date	Collection site	Collector	SSU	LSU	tef	RPB1	RPB2
**JPMA106**	*Salganea esakii* (teleomorph)	22-Jun-2019	Shirakawayama, Issou, Yakushima, Kagoshima	Kinjo N.	LC590837	N.A.	N.A.	N.A.	N.A.
**JPMA107**	*Salganea esakii* (anamorph)	22-Jun-2019	Shirakawayama, Issou, Yakushima, Kagoshima	Kinjo, N.	MT741703	MT741716	MT759574	MT759577	N.A.
**JPMA124**	*Salganea taiwanensis* (teleomorph)	21-Jun-2017	Yonahadake, Kunigami, Okinawa	Moriguchi, M. G.	MT741702	MT741717	MT759573	N.A.	N.A.
**JPMA125**	*Salganea esakii* (immature)	27-Jun-2004	Mukabakiyama, Nobeoka, Miyazaki	Kurogi, S.	N.A.	LC590838	N.A.	N.A.	N.A.
**JPMA135**	*Salganea esakii* (teleomorph)	27-Jun-2004	Mukabakiyama, Nobeoka, Miyazaki	Kurogi, S.	N.A.	N.A.	N.A.	N.A.	N.A.
**Moriguchi01**	*Salganea taiwanensis*	30-Apr-2019	Yonahadake, Kunigami, Okinawa	Moriguchi, M. G.	MT741705	MT741719	MT759575	MT759578	MT759580
**Moriguchi02**	*Salganea taiwanensis*	12-May-2019	Yonahadake, Kunigami, Okinawa	Moriguchi, M. G.	MT741704	MT741718	MT759572	MT759579	MT759581
**Moriguchi04**	*Salganea taiwanensis*	29-May-2016	Kunigami-son, Okinawa	Moriguchi, M. G.	MT741706	MT741720	MT759576	N.A.	N.A.

### Morphological studies

**Fig. 4:**
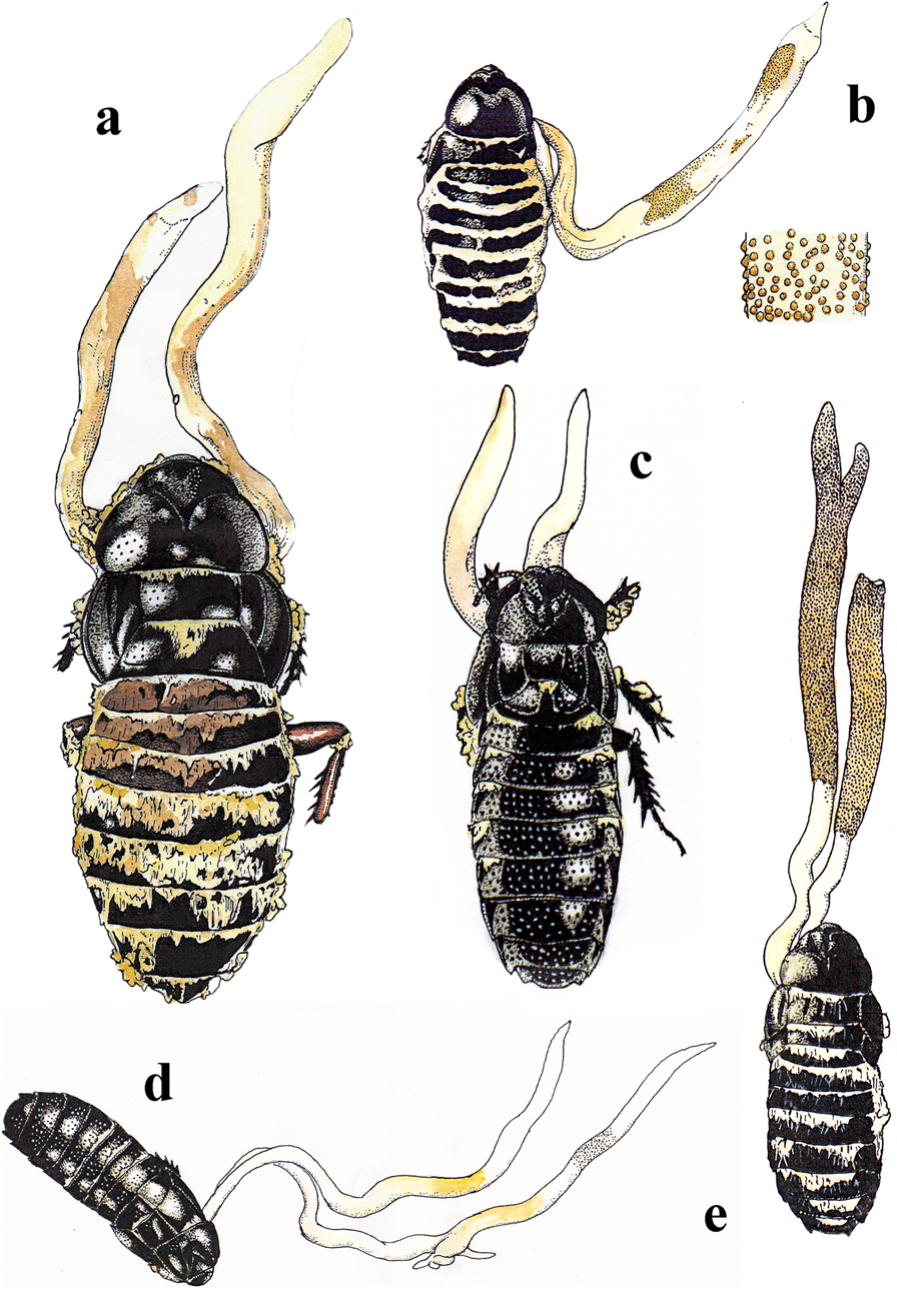
*Ophiocordyceps salganeicola* at various stages of development (del. M. G. Moriguchi). **a** Sexual morph of *O. salganeicola* growing on *Salganea esakii* collected in Miyazaki, 22 Jun. 2005. **b** Sexual morph on *S. esakii* collected in Yakushima, 17 Jun. 2017. with a close-up illustration of perithecia. **c** Early-stage specimen. **d** Mid-stage specimen (Mori04; see Tables 1 and 2) collected in Katsuu-dake, Nago-shi, Okinawa, 23 Apr. 2016. **e** Mature specimen from Kunigami-son, Okinawa, collected 29 May 2016

For macro-morphological characterization, specimens were examined using a stereoscopic microscope (Leica S8 APO) and sorted for further micro-morphological investigation. The characters investigated were ascomatal size, color, position, presence/absence and characterization of asexual morphs and perithecial insertion (e.g. immersed, semi-immersed, erumpent, superficial). For micro-morphological characterization, either free-hand or cryo-sectioning of the ascoma was performed using a Leica CM1850 Cryostat. Samples were mounted on a slide with plain lactic acid or lacto-fuchsin (0.1 g of acid fuchsin in 100 mL of lactic acid) for light microscopy examination using a Nikon Eclipse Ni-U. A minimum of 50 ascospores were measured for morphological comparison. The illustrations of fungal specimens were drawn based on the observation of photographs using drawing pens 0.13 mm and 0.2 mm (Rotring, Hamburg, Germany), painted by watercolors (HOLBEIN Art Materials Inc., Osaka, Japan) and scanned for imaging (Fig. 4). We also present the morphological comparison between *Ophiocordyceps* species infecting cockroaches and termites (Table 2).

**Table 2:** Morphological and ecological comparison between *Ophiocordyceps* species associated with blattodean insects (termites and cockroaches)

Species	Stromata	Perithecia	Asci	Ascospores	Distribution	Death position	Host	Reference
*Ophiocordyceps asiatica*	Solitary, simple, filiform, up to 15 cm long, orange-brown	Superficial, globose to sub-globose, 240–320 × 180–260 μm	filiform 92.5–175 × 5–6.3 μm	whole, septate, 90–132.5 × 1–2 μm	Thailand	Buried in soil	Blattodea, Isoptera, Termitidae, Macrotermitinae (Reproductive caste)	Tasanathai et al. (2019)
*Ophiocordyceps brunneirubra*	Solitary, simple or branched, narrowly clavate, slender and wiry, 9.5 cm long, orange brown to red brown	Immersed, ovoid, 300–400 × 130–200 μm	Cylindrical, 155–225 × 4.5–8 μm	Whole, filiform, septate, 156.5–197.5 × 2–3 μm	Thailand	Buried in soil	Blattodea, Isoptera, Termitidae, Macrotermitinae (Reproductive caste)	Tasanathai et al. (2019)
*Ophiocordyceps khokpasiensis*	Solitary, simple, cylindrical 16 cm long, brown	pseudo-immersed, sub-globose 200–250 × 120–200 μm	Filiform, 62.5–125 × 4–5 μm	Whole, filiform, 46–90 × 2–3 μm	Thailand	Buried in soil	Blattodea, Isoptera, Termitidae, Macrotermitinae (Reproductive caste)	Tasanathai et al. (2019)
*Ophiocordyceps mosingtoensis*	Solitary, simple, cylindrical, 11 cm long, brown to grey	Pseudo-immersed, ovoid, 400–500 × 200–300 μm	Filiform, 187.5–287.5 × 4.5–7.5 μm	Whole, filiform, septate, 230–315 × 1.5–3 μm	Thailand	Buried in soil	Blattodea, Isoptera, Termitidae, Macrotermitinae (Reproductive caste)	Tasanathai et al. (2019)
*Ophiocordyceps pseudocommunis*	Solitary, simple, cylindrical, up to 21 cm long, yellow brown	Superficial, sub-globose 520–600 × 360–440 μm	Filiform, 160–165 × 14–17	Whole with 7–8 septa, 107.5–147.5 × 6–7.5	Thailand	Buried in soil	Blattodea, Isoptera, Termitidae, Macrotermitinae (Reproductive caste)	Tasanathai et al. (2019)
*Ophiocordyceps communis*	Solitary, simple, filiform, 5–13 cm long, yellow brown	Superficial 285–675 × 195–390 μm	Filiform, 215–250 × 15 μm	Whole, filiform, 100–180 × 5–6 μm	Thailand	Soil	Blattodea, Isoptera	Sung et al. (2007)
*Ophiocordyceps pseudorhizoidea*	Solitary, simple, filiform, up to 21 cm long, light brown	Superficial, ovoid, 280–390 × 160–220 μm	Cylindrical, 120–150 × 5–7 μm	Whole, septate, 65–82.5 × 2–3 μm	Thailand	Buried in soil	Blattodea, Isoptera, Termitidae, Macrotermitinae (Reproductive caste)	Tasanathai et al. (2019)
*Ophiocordyceps termiticola*	Solitary, simple, filiform, up to 14 cm long, yellow brown	Pseudo-immersed, globose to sub-globose, 200–280 × 150–250 μm	Filiform, 62.5–110 × 4–6 μm	Whole, filiform, 85 × 2 μm	Thailand	Buried in soil	Blattodea, Isoptera, Termitidae, Macrotermitinae (Reproductive caste)	Tasanathai et al. (2019)
*Ophiocordyceps blattae*	Solitary, cylindric, 1 cm long, grey to lavender (“tissue” dark red-brown)	Immersed, conoid, 200 × 150 μm	Cylindrico–clavate, 4 or 8-spored 100–130 × 8–12 μm	Whole, multi-septate, elongated-fusoid, 50–80 × 3–4 μm	Sri Lanka	Underside of leaves	Blattodea, Blattidae	Petch (1924)
***Ophiocordyceps salganeicola***	**One or two, clavate to cylindrical, 1–7 × 0.15 cm, cream to brown**	**Immersed to semi-immersed, ovoid to flask-shaped, (325–) 365 (− 408) × 100–140 μm**	**Elongated clavate, hyaline, 8-spored, 150–200 × 7–11 μm**	**Whole, hyaline, 70–100 × 3 μm, 7-septate**	**Japan**	**Inside dead wood**	**Blattodea, Blaberidae, Panesthiinae (adult of** ***Salganea esakii*,** ***S. taiwanensis*)**	**This study**
*Ophiocordyceps blattarioides*	Gregarious, Simple, capitate, fertile part ovoid to sub-globoid, chestnut brown, 1.4–2 cm long	Immersed, ellipsoid, (650–) 760–800 × 220–300 μm	Cylindrical, (180–) 250 (−300) × 4–5 μm	Partspores, truncate, 6–12 (− 16) × 1.5 μm	Belize, Colombia, Ecuador	Leaf litter	Blattodea, Blattidae, Dictyoptera (adult of *Neostylopyga* sp.)	Sanjuan et al. (2015)
*Ophiocordyceps bispora*	Multiple (20–30), clavate, simple of branched, cream with dark perithecia, 1.5 × 0.15 cm	Globose, 300–375 × 375 μm	Clavate, 2-spored, 162–163 × 58–61 μm	Eliptical, flattened in one side, thick walled, dark, 95–105 × 34–35.4 μm	Tanzania, Kenya	Underneath stone	Blattodea, Isoptera, Termitidae, Macrotermitidae, (*Macrotermes natalensis, M. subhyalinus*, M. *michealseni*)	Stifler 1941; Ochiel et al. (1997)
*Ophiocordyceps octospora*	Multiple, clavate, 0.2–0.3 cm long	Sub-globose to ovoid, 180–220 × 200 μm	Clavate, 8-spored, 250 × 60 μm	Cylindric, flattened in one side curved on the other, no septa, 40–70 × 15–30 μm	Mexico	Near a stone wall	Blattodea, Isoptera, Termitidae (*Tenuirostitermes tenuirostris*)	Blackwell and Gilbertson (1981)

### DNA extraction, PCR and sequencing

All specimens used in this study were collected in their natural habitat. The material was preserved either dried or in ethanol and DNA extractions were performed with the following protocol: Parts of fungal tissues were removed from the host, placed in 1.5 ml Eppendorf tubes with 100–200 μl of CTAB readily after its collection and stored at room temperature, or entire samples were immersed in 70% ethanol and stored in the freezer. For DNA extraction, the samples were ground mechanically with 400 μl of CTAB and incubated at 60 °C for 20 min and centrifuged for 10 min at 14,000 rpm. The supernatant (approx. 400 μl) was transferred to a new 1.5 ml Eppendorf tube, mixed with 500 μl of 24:1 chloroform: isoamyl-alcohol (FUJIFILM Wako Pure Chemical Corp., Osaka, Japan) and mixed by inverting. The mix was then centrifuged for 20 min at 14,000 rpm and the supernatant transferred to a new 1.5 ml Eppendorf tube and further cleaned using the GeneCleanIII kit (MP Biomedicals, Santa Ana, CA, USA), following the recommended protocol. The only step modified was the addition of 30 μl of GlassMilk per sample, instead of the recommended 10 μl, aiming to increase yield.

Five loci were used in the analyses, i.e. small subunit nuclear ribosomal DNA (SSU), large subunit nuclear ribosomal DNA (LSU), translation elongation factor 1-α (TEF) and the largest and second largest subunits of RNA polymerase II (RPB1 and RPB2 respectively) with a total read length of 4815 bp. The primers used were, SSU: 82F (5′-GAAACTGCGAATGGCT-3′) and 1067R (5′-TMTCGTAAGGTGCCGA-3′) (Matsuura et al. 2018); LSU: LR0R (5′-ACCCGCTGAACTTAAGC-3′) and LR5 (5′-TCCTGAGGGAAACTTCG-3′) (Vilgalys and Sun 1994); TEF: 983F (5′-GCYCCYGGHCAYCGTGAYTTYAT-3′) and 2218R (5′- ATGACACCRACRGCRACRGTYTG-3′); cRPB1: (5′-CCWGGYTTYATCAAGAARGT-3′) and RPB1Cr (5′-CCNGCDATNTCRTTRTCCATRTA-3′) (Castlebury et al. 2004). RPB2: fRPB2-5F:(5′-GAYGAYMGWGATCAYTTYGG-3′) and fRPB2-7cR (5′- CCCATRGCTTGTYYRCCCAT − 3′) (Liu et al. 1999).

Each 20 μl-PCR reaction contained 10 μl of Ampdirect® Plus 2x (Shimazu Corp., Kyoto, Japan), 0.6 μl of each forward and reverse primers (10 mM), 1 μl of DNA template, 0.1 TaKaRa *Ex Taq* DNA Polymerase (Takara Bio Inc., Kusatsu, Shiga, Japan) and 8.7 μl of Ultra Pure Distilled Water (Thermo Fisher Scientific Inc., Waltham, MA, USA). The PCR reactions were placed in an Astec PC-818 thermocycler under the following conditions: for SSU and LSU (1) 2 min at 95 °C, (2) 10 cycles of denaturation at 95 °C for 30 s, annealing at 62 °C for 30 s, and extension at 72 °C for 2 min, followed by (3) 25 cycles of denaturation at 95 °C for 30 s, annealing at 55 °C for 30 s, and extension at 72 °C for 2 min and (4) 3 min at 72 °C. For TEF and RPB1(1) 2 min at 95 °C, (2) 10 cycles of denaturation at 95 °C for 30 s, annealing at 60 °C for 40 s, and extension at 72 °C for 1 min 30 s, followed by (3) 30 cycles of denaturation at 95 °C for 30 s, annealing at 55 °C for 40 s, and extension at 72 °C for 1 min 30 s and (4) 3 min at 72 °C. Each PCR reaction was partially electrophoresed and the rest was cleaned by adding 3.0 μl of enzymatic PCR clean-up reagent, consisting of 0.1 μl of Exonuclease I (New England BioLabs, Ipswich, MA, USA) and 0.1 μl of alkaline phosphatase (shrimp) (Takara Bio Inc., Kusatsu, Shiga, Japan), incubated at 37 °C for 20 min and 80 °C for 15 min in the thermocycler. The processed PCR products were directly sequenced by a capillary DNA sequencer, Genetic Analyzer 3130xl (Thermo Fisher Scientific Inc., Waltham, MA, USA) at C-RAC of the University of the Ryukyus.

### Phylogenetic analyses

The raw sequence reads (.ab1 files) were edited manually using Geneious 11.1.5 (https://www.geneious.com). Individual gene alignments were generated by MAFFT (Katoh and Standley 2013). The alignment of every gene was improved manually, annotated and concatenated into a single combined dataset using Geneious 11.1.5. Ambiguously aligned regions were excluded from phylogenetic analysis and gaps were treated as missing data. The final alignment length was 4629 bp: 1020 bp for SSU, 870 bp for LSU, 967 bp for TEF, 683 bp for RPB1 and 1089 for RPB2. Maximum likelihood (ML) analysis was performed with RAxML version 8.2.4 (Stamatakis 2014) on a concatenated dataset containing all five genes. The dataset consisted of 11 data partitions, 2 for SSU and LSU, and 9 for each codon position of the three protein coding genes: TEF, RPB1 and RPB2. The GTRGAMMA model of nucleotide substitution was employed during the generation of 1000 bootstrap replicates. The sequences for all *Ophiocordyceps* used in this study are presented in Table 3.

**Table 3:** Genbank accession number, host association and reference for all *Ophiocordyceps* species used in this study

Species	Voucher #	SSU	LSU	TEF	RPB1	RPB2	Host order	Reference
*Hirsutella cryptosclerotium*	ARSEF 4517	–	KM652109	KM651992	KM652032	–	Hemiptera (Pseudococcidae	Tasanathai et al. (2019)
*Hirsutella necatrix*	ARSEF 5549	–	KM652116	KM651999	KM652039	–	Acari (Eriophydae)	Tasanathai et al. (2019)
*Hirsutella thompsonii var. vinacea*	ARSEF 254	–	KM652149	KM652028	KM652062	–	Acari (Eriophydae)	Tasanathai et al. (2019)
*Hymenostilbe aurantiaca*	OSC128578	DQ522556	DQ518770	DQ522345	DQ522391	DQ522445	Hymenoptera	Quandt et al. (2014)
*Ophiocordyceps acicularis*	OSC 128580	DQ522543	DQ518757	DQ522326	DQ522371	DQ522423	Coleoptera	Quandt et al. 2014
*Ophiocordyceps agriotidis*	ARSEF 5692	DQ522540	DQ518754	DQ522322	DQ522368	DQ522418	Coleoptera	Quandt et al. 2014
*Ophiocordyceps albacongiuae*	RC20	KX713633	–	KX713670	–	–	Hymenoptera	Araújo et al. (2019)
*Ophiocordyceps annulata*	CEM303	KJ878915	KJ878881	KJ878962	KJ878995	–	Coleoptera	Quandt et al. (2014)
*Ophiocordyceps aphodii*	ARSEF 5498	DQ522541	DQ518755	DQ522323	–	DQ522419	Coleoptera	Quandt et al. (2014)
*Ophiocordyceps aphodii*	ARSEF 5498	DQ522541	DQ518755	DQ522323	–	DQ522419	Coleoptera	Quandt et al. (2014)
*Ophiocordyceps asiatica*	BCC 30516	–	MH753675	MK284263	MK214105	MK214091	Blattodea (Termitidae)	Tasanathai et al. (2019)
*Ophiocordyceps australis*	HUA 186097	KC610786	KC610765	KC610735	KF658662	–	Hymenoptera	Sanjuan et al. (2015)
*Ophiocordyceps australis*	HUA 186147	KC610784	KC610764	KC610734	KF658678	–	Hymenoptera	Quandt et al. 2014
*Ophiocordyceps bispora*	KVL 606	AH006986	AF009654	–	–	–	Blattodea (Termitidae)	Conlon et al. (2017)
*Ophiocordyceps blakebarnesii*	MISSOU5	KX713641	KX713610	KX713688	KX713716	–	Hymenoptera	Araújo et al. (2018)
***Ophiocordyceps*** **cf.** ***blattae***	**BCC38241**	**–**	**MT512657**	**MT533485**	**MT533479**	**–**	**Blattodea (Blattoidea)**	**This study**
*Ophiocordyceps brunneipunctata*	OSC 128576	DQ522542	DQ518756	DQ522324	DQ522369	DQ522420	Coleoptera	Quandt et al. (2014)
*Ophiocordyceps brunneirubra*	BCC 14384	–	MH753690	GU797121	MK751465	MK751468	Blattodea	Tasanathai et al. (2019)
*Ophiocordyceps buquetii*	HMAS_199613	KJ878939	KJ878904	KJ878984	KJ879019	–	Hymenoptera	Araújo et al. (2018)
*Ophiocordyceps camponoti-balzani*	G104	KX713660	KX713593	KX713689	KX713703	–	Hymenoptera	Araújo et al. (2018)
*Ophiocordyceps camponoti-bispinosi*	OBIS5	KX713636	KX713616	KX713693	KX713721	–	Hymenoptera	Araújo et al. (2018)
*Ophiocordyceps camponoti-femorati*	FEMO2	KX713663	KX713590	KX713678	KX713702	–	Hymenoptera	Araújo et al. (2018)
*Ophiocordyceps camponoti-hippocrepidis*	HIPPOC	KX713655	KX713597	KX713673	KX713707	–	Hymenoptera	Araújo et al. (2018)
*Ophiocordyceps camponoti-nidulantis*	NIDUL2	KX713640	KX713611	KX713669	KX713717	–	Hymenoptera	Araújo et al. (2018)
*Ophiocordyceps camponoti-rufipedis*	G108	KX713659	KX713594	KX713679	KX713704	–	Hymenoptera	Araújo et al. (2018)
*Ophiocordyceps cochlidiicola*	HMAS_199612	KJ878917	KJ878884	KJ878965	KJ878998	–	Lepidoptera	Quandt et al. 2014
*Ophiocordyceps communis*	BCC 1842	–	MH753680	MK284266	MK214110	MK214096	Blattodea (Termitidae)	Tasanathai et al. (2019)
*Ophiocordyceps curculionum*	OSC 151910	KJ878918	KJ878885	–	KJ878999	–	Coleoptera	Quandt et al. (2014)
*Ophiocordyceps diabolica*	BDS 32	MK393830	MK393322	–	–	–	Hymenoptera	Araújo et al. (2018)
*Ophiocordyceps elongata*	OSC 110989	–	EF468808	EF468748	EF468856	–	Lepidoptera	Quandt et al. 2014
*Ophiocordyceps evansii*	HUA 186159	KC610796	KC610770	KC610736	KP212916	–	Hymenoptera	Sanjuan et al. (2015)
*Ophiocordyceps formicarum*	TNS F18565	KJ878921	KJ878888	KJ878968	KJ879002	KJ878946	Hymenoptera	Quandt et al. 2014
*Ophiocordyceps formosana*	TNM F13893	KJ878908	–	KJ878956	KJ878988	KJ878943	Coleoptera	Quandt et al. (2014)
*Ophiocordyceps forquignonii*	OSC 151908	KJ878922	KJ878889	–	KJ879003	KJ878947	Diptera	Quandt et al. 2014
*Ophiocordyceps fulgoromorphila*	HUA 186139	KC610794	KC610760	KC610729	KF658676	KC610719	Hemiptera	Sanjuan et al. (2015)
*Ophiocordyceps fulgoromorphila*	HUA 186142	KC610795	KC610761	KC610730	KF658677	–	Hemiptera	Sanjuan et al. (2015)
*Ophiocordyceps gracillima*	HUA 186132	–	KC610768	KC610744	KF658666	–	Coleoptera	Sanjuan et al. (2015)
*Ophiocordyceps humbertii*	MF116b	MF116B	MK874748	MK875536	–	MK863828	Hymenoptera	Araújo et al. (2019)
*Ophiocordyceps irangiensis*	OSC 128577	DQ522546	DQ518760	DQ522329	DQ522374	DQ522427	Hymenoptera	Sanjuan et al. (2015)
*Ophiocordyceps khokpasiensis*	BCC 48071	–	MH753682	MK284269	MK214112	–	Blattodea (Termitidae)	Tasanathai et al. (2019)
*Ophiocordyceps kimflemingiae*	SC30	KX713629	KX713622	KX713699	KX713727	–	Hymenoptera	Araújo et al. (2019)
*Ophiocordyceps lloydii*	OSC 151913	KJ878924	KJ878891	KJ878970	KJ879004	KJ878948	Hymenoptera	Quandt et al. (2014)
*Ophiocordyceps longissima*	HMAS_199600	KJ878926		KJ878972	KJ879006	KJ878949	Hemiptera	Quandt et al. (2014)
*Ophiocordyceps melolonthae*	OSC 110993	DQ522548	DQ518762	DQ522331	DQ522376	–	Coleoptera	Quandt et al. (2014)
*Ophiocordyceps mosingtonensis*	BCC 30904	–	MH753686	MK284273	MK214115	MK214100	Blattodea (Termitidae)	Tasanathai et al. (2019)
*Ophiocordyceps myrmecophila*	HMAS_199620	KJ878927	KJ878893	KJ878973	KJ879007	–	Hymenoptera	Quandt et al. (2014)
*Ophiocordyceps neovolkiana*	OSC 151903	KJ878930	KJ878896	KJ878976	KJ879010	–	Coleoptera	Quandt et al. (2014)
*Ophiocordyceps nigrella*	EFCC 9247	EF468963	EF468818	EF468758	EF468866	EF468920	Coleoptera	Quandt et al. (2014)
*Ophiocordyceps nutans*	OSC 110994	DQ522549	DQ518763	DQ522333	DQ522378	–	Hemiptera	Quandt et al. (2014)
*Ophiocordyceps ootakii*	J13	KX713652	KX713600	KX713681	KX713708	–	Hymenoptera	Araújo et al. (2018)
*Ophiocordyceps palthothyreum*	Palt1	MK393848	MK393345	–	–	–	Hymenoptera	Araújo et al. (2019)
*Ophiocordyceps pruinosa*	NHJ 12994	EU369106	EU369041	EU369024	EU369063	EU369084	Lepidoptera	Quandt et al. (2014)
*Ophiocordyceps pseudocommunis*	BCC 16757	–	MH753687	MK284274	MK214117	MK214101	Blattodea (Termitidae)	Tasanathai et al. (2019)
*Ophiocordyceps pseudorhizoidea*	BCC 48879	–	MH753673	MK284261	MK214104	MK214089	Blattodea (Termitidae)	Tasanathai et al. (2019)
*Ophiocordyceps pulvinata*	TNS-F 30044	GU904208	–	GU904209	GU904210	–	Hymenoptera	Kepler et al. (2011)
*Ophiocordyceps purpureostromata*	TNS F1843	KJ878931	KJ878897	KJ878977	KJ879011	–	Coleoptera	Quandt et al. (2014)
*Ophiocordyceps ravenelii*	OSC 110995	DQ522550	DQ518764	DQ522334	DQ522379	DQ522430	Coleoptera	Quandt et al. (2014)
*Ophiocordyceps rhizoidea*	NHJ 12522	EF468970	EF468825	EF468764	EF468873	EF468923	Coleoptera	Quandt et al. (2014)
***Ophiocordyceps salganeicola***	**JPMA107**	**MT741703**	**MT741716**	**MT759574**	**MT759577**	**–**	**Blattodea (Blattoidea)**	**This study**
***Ophiocordyceps salganeicola***	**JPMA124**	**MT741702**	**MT741717**	**MT759573**	**–**	**–**	**Blattodea (Blattoidea)**	**This study**
***Ophiocordyceps salganeicola***	**Mori01**	**MT741705**	**MT741719**	**MT759575**	**MT759578**	**MT759580**	**Blattodea (Blattoidea)**	**This study**
***Ophiocordyceps salganeicola***	**Mori02**	**MT741704**	**MT741718**	**MT759572**	**MT759579**	**MT759581**	**Blattodea (Blattoidea)**	**This study**
***Ophiocordyceps salganeicola***	**Mori04**	**MT741706**	**MT741720**	**MT759576**	**–**	**–**	**Blattodea (Blattoidea)**	**This study**
*Ophiocordyceps satoi*	J7	KX713653	KX713599	KX713683	KX713711	–	Hymenoptera	Araújo et al. (2018)
*Ophiocordyceps sinensis*	EFCC 7287	EF468971	EF468827	EF468767	EF468874	EF468924	Lepidoptera	Quandt et al. (2014)
*Ophiocordyceps sobolifera*	KEW 78842	EF468972	EF468828	–	EF468875	EF468925	Hemiptera	Quandt et al. (2014)
*Ophiocordyceps sp.*	OSC 151909	KJ878936	KJ878900	KJ878982	KJ879016	KJ878952	Hymenoptera	Quandt et al. (2014)
*Ophiocordyceps sphecocephala*	OSC 110998	DQ522551	DQ518765	DQ522336	DQ522381	DQ522432	Hymenoptera	Quandt et al. (2014)
*Ophiocordyceps stylophora*	OSC 111000	DQ522552	DQ518766	DQ522337	DQ522382	DQ522433	Coleoptera	Quandt et al. (2014)
*Ophiocordyceps termiticola*	BCC 1920	–	MH753678	MK284265	MK214108	MK214094	Blattodea (Termitidae)	Tasanathai et al. (2019)
*Ophiocordyceps variabilis*	OSC 111003	EF468985	EF468839	EF468779	EF468885	EF468933	Coleoptera	Quandt et al. (2014)
*Ophiocordyceps yakusimensis*	HMAS_199604	KJ878938	KJ878902	–	KJ879018	KJ878953	Hemiptera	Quandt et al. (2014)
*Paraisaria amazonica*	HUA 186113	KJ917566	KJ917571		KP212903	KM411980	Orthoptera	Sanjuan et al. (2015)
*Paraisaria blattarioides*	HUA186093	KJ917559	KJ917570	KM411992	KP212910	–	Blattodea (Blattoidea)	Sanjuan et al. (2015)
*Paraisaria blattarioides*	HUA 186108	KJ917558	KJ917569	–	KP212912	KM411984	Blattodea (Blattoidea)	Sanjuan et al. (2015)
*Paraisaria gracilis*	EFCC 8572	EF468956	EF468811	EF468751	EF468859	EF468912	Lepidoptera	Quandt et al. 2014
*Paraisaria heteropoda*	EFCC 10125	EF468957	EF468812	EF468752	EF468860	EF468914	Hemiptera	Quandt et al. 2014
*Paraisaria sp.*	OSC 151904	KJ878934	KJ878899	KJ878980	KJ879014	–	Hemiptera	Quandt et al. (2014)
*Paraisaria sp.*	OSC 151905	KJ878935	–	KJ878981	KJ879015	KJ878951	Hemiptera	Quandt et al. (2014)

### Ancestral character state reconstruction (ACSR)

**Fig. 5:**
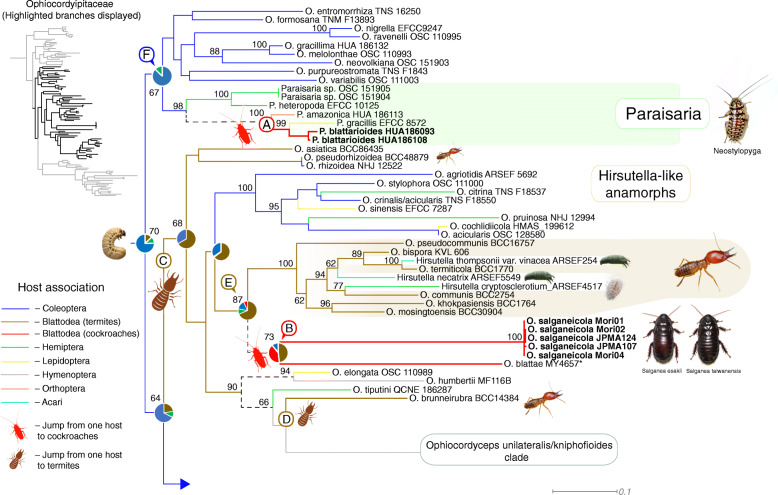
Maximum likelihood tree of *Ophiocordycipitaceae* obtained from RAxML analyses based on a concatenated set of 5 genes (SSU, LSU, TEF, RPB1 and RPB2). Colored branches reflect Ancestral Character State Reconstruction (ACSR) analyses based on host associations (See legend at the bottom-left) and pie-charts represent the probability for the association with host orders. Dashed lines indicate ambiguous association. Host pictures by Alex Wild and Shizuma Yanagisawa

To understand the evolutionary pathways of host association of *Ophiocordyceps* and blattodean insects, we conducted ancestral character reconstruction in Mesquite (Maddison and Maddison 2018) of the whole genus, using the best-scoring ML tree produced in RAxML (Stamatakis 2014). We coded each taxon based on host association (8 categories: *Acari, Coleoptera, Hymenoptera, Lepidoptera, Hemiptera, Orthoptera* and *Blattodea* divided into cockroaches and termites – Fig. 5). We used maximum likelihood model MK1, as implemented in Mesquite 3.51 (Maddison and Maddison 2018). Only nodes presenting > 70% of probability were displayed and used to color-code the branches on the Fig. 5. Nodes below this limit were treated as ambiguous and displayed as dashed lines.

## TAXONOMY

***Ophiocordyceps salganeicola*** Araújo, Moriguchi & Matsuura**, sp. nov.**

(Figs. 6, 7 and 8)

**Fig. 6:**
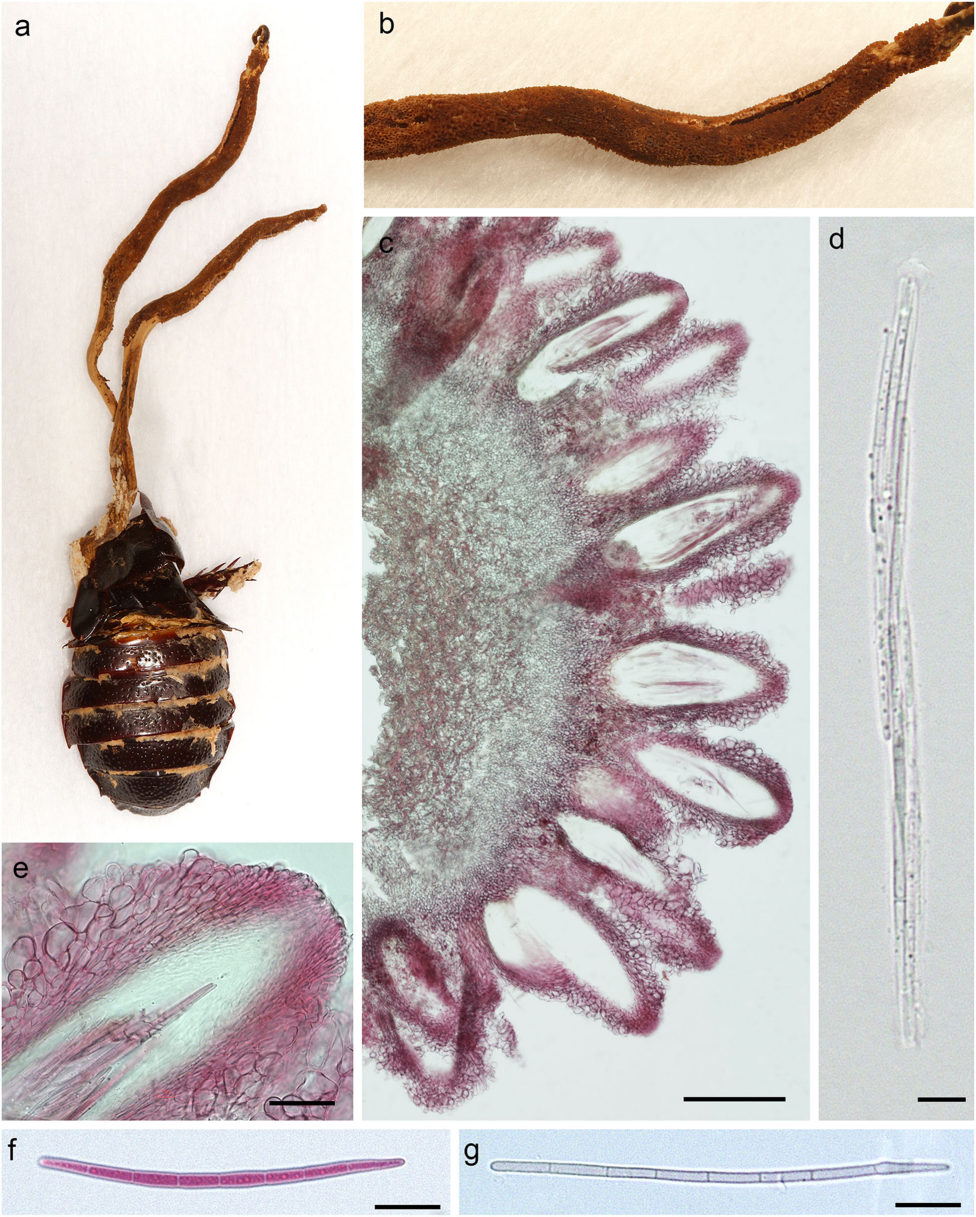
*Ophiocordyceps salganeicola* on *Salganea taiwanensis* (dried specimen) from Kunigami-son, Okinawa (TNS-F-60532). **a** Two ascomata arising from *S. taiwanensis*. **b** Close-up of dried ascoma. **c** Cross section of ascoma showing the perithecial arrangement. **d** Ascus with spirally twisted ascospores. **e** Perithecial ostiole. **f** and **g** 8-celled ascospore. Scale bars = **c** 200 μm, **d** 15 μm, **e** 10 μm, **f** and **g** 20 μm. Presented as JPMA124 in

**Fig. 7:**
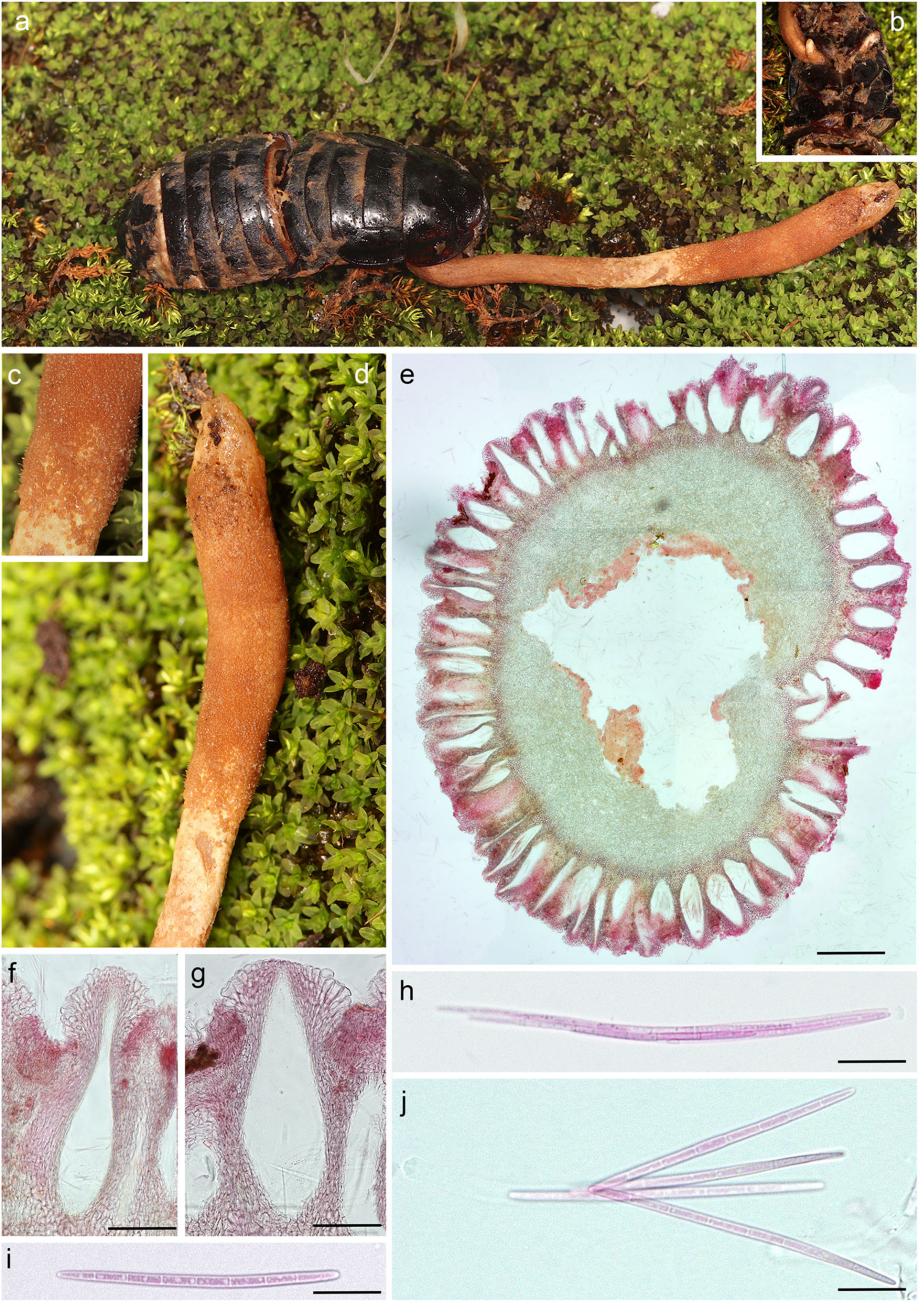
*Ophiocordyceps salganeicola* on *Salganea esakii* (fresh specimen) from Yakushima, Kagoshima (TNS-F-91239). **a** *Salganea esakii* with a single robust ascoma; **b** Close-up showing early stage ascoma arising from ventral pronotum; **c** and **d** Close-up of ascoma; **e** Cross-section of ascoma; **f** and **g** Perithecia; **h** Ascospores within ascus; **i** and **j** 8-celled ascospores. Presented as JPMA106 in Fig. 5

**Fig. 8:**
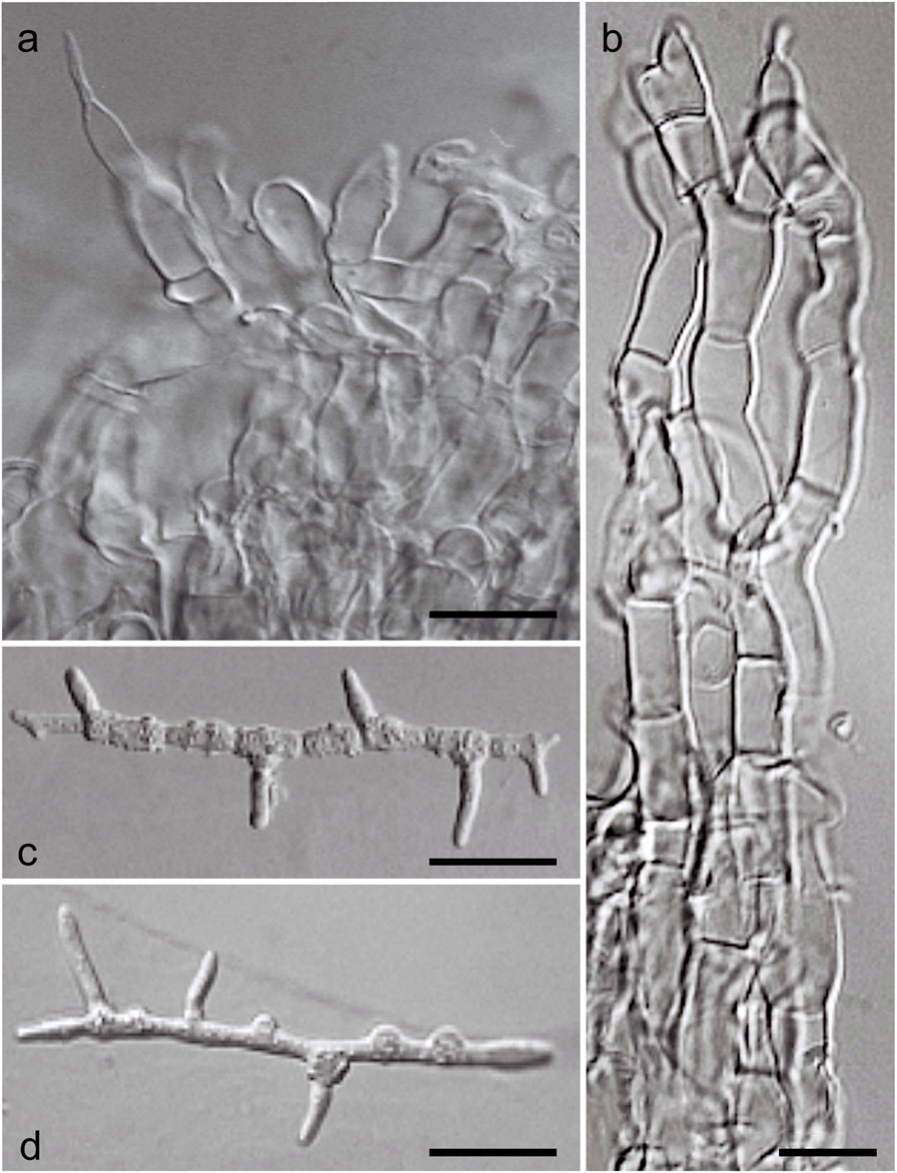
*Ophiocordyceps salganeicola* anamorph and germinated ascospores. **a** and **b** *Hirsutella*-like conidiogenous cells, **c** and **d** Ascospores germination after 72 h

Mycobank MB836091.

*Etymology*: Named after the host genus *Salganea*.

*Diagnosis*: *Ophiocordyceps salganeicola* can be easily differentiated from other closely-related species by its unique host association, ascomatal morphology and its strict distribution across the Southern Islands of Japan. Other closely related species are associated with termites, mites and hemipterans and exhibit completely different macro morphology, being easily distinguished still in the field.

*Type*: **Japan**: *Okinawa*: Kunigami-son, Yonahadake, 26°43′45.0″N 128°12′48.2″E, on *Salganea taiwanensis* (*Blattodea*, *Blaberidae*), 21 June 2017, *M.G. Moriguchi* (TNS-F-60532 – holotype).

*Description*: *External mycelium* sparse, light to dark brown, arising from the host’s sutures. *Stromata* one or two, 1–7 cm long, 1.3–5 mm thick, cream to light or dark brown, clavate to cylindrical in shape. *Perithecia* immersed, usually covering the apical part descending tol about the middle of the stromata, immersed to semi-immersed, ovoid to flask-shaped, (325–) 365 (− 408) × 100–140 μm. *Asci* hyaline, elongated clavate, 150–200 × 7–11 μm with prominent cap, 8-spored. *Ascospores* hyaline, 70–100 × 3 μm, 7-septate, not disarticulating into part-spores. *Asexual morph:* hirsutella-like phialides occurring sparsely on the surface of the stromata where perithecia are absent, 7–16 × 6–8 μm with neck measuring 18–30 × 5–7.5. *Conidia*: ovoid, 7 × 5 μm, hyaline to pale brown.

*Host*: *Salganea taiwanesnsis* and *S. esakii*.

*Habitat*: Forests of Miyazaki Prefecture, Yakushima and Okinawa islands of Japan. On remains of the hosts inside rotten logs, with only the fungus sporophores emerging.

*Distribution*: Only currently known from Japan.

*Other specimen examined*: **Japan**: Shirakawayama, Issou, *Yakushima*, Kagoshima 30°20′58.0″N 130°36′35.”E, on *Salganea esakii* (*Blattodea*, *Blaberidae*), 22 June 2019, *N. Kinjo* (TNS-F-91239 – paratype) (as JPMA106 see Fig. 7).

## RESULTS

### Molecular phylogeny and evolutionary origins of cockroach-associated *Ophiocordyceps*

We obtained 20 new sequences from five specimens of *O. salganeicola* (Fig. 3, Table 3). Our phylogenetic analysis is in accordance with previously published *Ophiocordyceps* topologies (Quandt et al. 2014; Sanjuan et al. 2015; Araújo et al. 2018; Tasanathai et al. 2019). All *O. salganeicola* specimens we collected, from different parts of Japan and infecting two species of *Salganea*, clustered together as a single species with a high degree of genetic similarity with a long branch (Fig. 5). It formed a monophyletic group with another cockroach-associated species, *O. blattae*, which is the type species for *Ophiocordyceps*. This is the first time *O.* cf. *blattae* is included in a phylogenetic study.

Our results indicate that *Ophiocordyceps* originated from a beetle-associated ancestor (72% ACSR), corroborating previous studies (Araújo and Hughes 2019). For the cockroach parasites, we found at least two independent origins within *Ophiocordyceps*, one within the *Paraisaria* clade, i.e. *Paraisaria blattarioides* (Fig. 5 node A), and the other within the hirsutelloid species, i.e. *O. salganeicola* and *O. blattae* (Fig. 5 node B). The ancestral host association for the cockroach-associated *Paraisaria* lineage was ambiguously recovered, while for the hirsutelloid cockroach-associated species our data show it has originated likely from a termite-associated ancestor, although this is not strongly supported (44% ACSR). We also found that the association with termites is older than cockroaches, evolving independently at least twice (Fig. 5 nodes C and D). The oldest, would have arisen from beetles to termites (65% ACSR, Fig. 5 node C). However, the origins of *O. brunneirubra* remains uncertain as part of the ancient termite-associated lineage (Fig. 5 node C) or if it jumped more recently from Hymenoptera to termites (Fig. 5 Node D).

The *Paraisaria* clade is an ecologically heterogeneous group composed of species parasitic on *Coleoptera*, *Orthoptera*, *Lepidoptera,* and *Hemiptera* (Mongkolsamrit et al. 2019). Our ACSR analysis provided weak resolution for the origins of *P. amazonica*/*P. blattarioides*/*P. gracilis* clade with 50.1% for *Orthoptera*, 25.9% for *Blattodea* (cockroaches) and 10.9% for *Lepidoptera* (Fig. 8 Node A). Our data also did not provide strong support for the ancestor of *P. blattarioides* with 51.1% for *Blattodea* (cockroaches), 21% for *Orthoptera* and 20.6% for *Lepidoptera* (Fig. 8 Node A). Nevertheless, the whole *Paraisaria* lineage was strongly supported as having evolved from a beetle parasite (Fig. 8 Node F, ACSR = 81.1%).

Conversely, for the novel clade composed of *O. salganeicola* and *O. blattae,* our results suggest (BS=87; ACSR=72.4%) that it evolved from an ancestral parasite on termites (Fig. 8 node E). *Ophiocordyceps salganeicola*/*blattae* was retrieved as a sister group to a clade composed mostly by termite (*Blattodea*, *Termitidae*) parasites with species associated with hemipterans (*Pseudococcidae*) and mites (*Acari*, *Eriophyidae*). According to our results, all host switches in this clade occurred from termites (i.e. termites to *Coleoptera*, termites to *Hemiptera*, termites to *Acari* and termites to cockroaches). Unexpectedly, our analyses also suggest (ACSR=61.7%) the clade composed by parasites of *Coleoptera*, *Lepidoptera,* and *Hemiptera*, including the economically and culturally important *O. sinensis*, could have originated from an ancestor infecting termites, instead of beetle larvae as previously proposed (Araújo and Hughes 2019).

## DISCUSSION

### Ecology and natural history of *Salganea–Ophiocordyceps* relationships

The insect cuticle represents a formidable barrier to infection by bacterial and fungal pathogens with relatively few having managed to cross it. Once inside the insect, innate immunity provides further challenges to an invading pathogen (Evans 1988). However, once these have been overcome the insect body provides a stable environment for their development. This is particularly true for colonial cockroaches that spend most of their lives protected inside nests, for example some wood-feeding species within the families *Cryptocercidae* and *Blaberidae*, specifically the subfamily *Panesthiinae* (*Panesthiini*, *Ancaudeliini*, *Caepariini,* and *Salganeini*). Among those groups, one of the most well-known social cockroaches is the genus *Salganea*, comprised of about 50 species (Beccaloni 2007; Bell et al. 2007; Wang et al. 2014). All the known species within the genus live within and feed on decaying wood, building chambers and galleries inside hardwood or coniferous logs that may take decades to degrade (Maekawa et al. 2008), providing long-term stable homeostatic conditions. Such a protected environment certainly benefits indirectly the fungal parasites that are already inside the host body. An exposed cadaver on the forest floor would be much more susceptible to being scavenged by animals or consumed by other microorganisms.

*Salganea* species form social groups, composed mostly of biparental families, consisting of a male-female and their offspring (Maekawa et al. 2008). Sociality endows insects with advantages such as increased efficiency of brood care, foraging and anti-predator defenses. However, infectious diseases can potentially spread more easily within a colony because of their high densities, frequent social contact and also because group members are often close relatives and thus susceptible to the same parasitic infections (Cremer et al. 2007). Therefore, it is surprising that only three species of *Ophiocordyceps*, a common and widespread genus of entomopathogenic fungi, have been recorded infecting the equally diverse and globally distributed cockroaches (Bourguignon et al. 2018). *Ophiocordyceps* species infecting social insects, notably ants, are one of the most broadly distributed and ubiquitous entomopathogenic fungi in tropical forests worldwide (Araújo et al. 2015, 2018). They often form epizootic events, in which hundreds of infected ants can be found in a small patch of forest (Evans and Samson 1982; Pontoppidan et al. 2009). On the other hand, however, *Ophiocordyceps* on social cockroaches are rare in Japan and only one or two infected individuals are collected in the same log, despite the ubiquity and abundance of hosts in one area.

Based on our extensive field surveys we found that ascomata of *O. salganeicola* in Okinawa start to emerge in decaying logs in early April. However, they seem to require at least a few months to become mature and develop the sexual morph in the field. This development occurs in parallel with the mating season of the host cockroaches from April to July when newly emerged adults leave their logs and parents, fly, mate and burrow into a new nest (Osaki Haruka, personal communication). Presumably, these young adults might become infected by the ascospores/conidia of *O. salganeicola* during colonization of a new log. The host is then later killed and consumed by the fungal parasite, eventually producing new fruiting bodies in the next mating season. On the other hand, there has been no record of an infected nymph in 26 fungal specimens observed in Okinawa, but only a single infected nymph (see Fig. 2e) out of more 27 specimens in Yakushima between 2015 and 2019, suggesting the outbreak of this fungus within an established colony is rather rare and the primary targets are likely newly emerged adults. However, this proposed life-cycle of *O. salganeicola* is only hypothetical and requires periodical field observations in the same ecological habitat along with host insect behaviors (Maekawa et al. 2008), in order to determine how and when the fungus infects and kills the host.

### Does *O. salganeicola* manipulate host behavior before death?

The behavior manipulation caused by *Ophiocordyceps* fungi on their hosts is a striking phenomenon, especially in species associated with ants, the so-called “zombie-ant fungi” (Evans et al. 2011; Araújo et al. 2018). It has been posited that species within the *Ophiocordyceps unilateralis* core clade infecting Camponotini ants evolved such an ability as a response to the strong social immunity displayed by ant societies that prevents fungal transmission and development inside the colony (Araújo and Hughes 2019). Conversely, as far as we know, there is no evidence of social cockroaches recognizing the infected members of their colony, except for the parental and sibling’s grooming behavior that might fend off superficial parasites. Thus, fungal infection, development and transmission could potentially occur in the same log where other members of the colony still inhabit, in which no drastic behavior manipulation is needed in order to remove the host from its nest and thus complete the parasite’s life-cycle. However, there is a possibility of a subtle manipulation.

*Salganea* cockroaches burrow and nest deep inside the trunk, only becoming exposed to the external environment in the mating season, whereas the *Ophiocordyceps*-infected ones are found only a few centimeters below the wood surface (Fig. 2). Thus, we posit that the fungus might potentially be able to manipulate host’s behavior by leading to a migration towards a more superficial layer inside the log. This host migration could be stimulated by the need for a more oxygen- and water-rich stratum and/or attraction to the light coming from an opening, through which the fungal ascoma emerge and disperse its spores (Fig. 2a–b). Further studies are needed to test this hypothesis.

### Host association, speciation, and distribution

While some distinct, mostly macro-, morphological features can be observed in *O. salganeicola* infecting both host species that diverged from a single ancestor around 4–5 mya (Maekawa et al. 1999; Maekawa and Matsumoto 2003), nucleotide sequences of fungal specimens are highly conserved among diverse strains in wide range of geographic regions and islands. The number of polymorphic sites within all aligned five gene sequences from multiple samples (Table 3) was only two out of 4202 and both were synonymous. Thus, geographic and reproductive isolation of the fungal strains may have not yet resulted in the allopatric speciation of *O. salganeicola.* The long branch length and high host specificity to the genus *Salganea* indicate a long co-evolutionary relationship with the host populations and hence unlikelihood of recent host jumping (Fig. 5 node B). However, we still do not know whether the single parasite strain can only persist in one geographic region and/or island infecting the same host populations over generations, or jump across multiple closely related host populations and species horizontally even after such a long geographic isolation of the Ryukyu Islands. In some of these islands such as Amamioshima and Tokunoshima in Kagoshima and Ishigaki-jima and Iriomote-jima in Okinawa, there has been no collection record of *O. salganeicola,* suggesting their current absence. These questions on the evolution of host associations of *O. salganeicola* in the Japanese archipelago of Ryukyus deserve particular attention for studying host-parasite co-evolution in the context of island biology, which possibly can be tested by artificial infection experiments using the field-collected fungal ascoma and laboratory-reared *Salganea* colonies from different islands. Furthermore, *S. taiwaensis* and the other *Salganea* cockroach species are distributed not only in Japan, but also in wide greographic regions in South, Southeast and East Asia (Wang et al. 2014). Additional screening of *Salganea* and related host species for entomopathogens in Asia-Pacific regions might unravel the phylogeography and evolutionary origins of cockroach-*Ophiocordyceps* associations.

### *Ophiocordyceps* cf. *blattae*

*Ophiocordyceps blattae* was described by Petch (1924) and was collected at Hakgala (Sri Lanka, formerly Ceylon) only twice (Fig. 1). A rare species, it was originally collected on cockroaches attached by fungal structures to the underside of leaves, exhibiting a cylindric, grey to lavender ascoma. The specimen we used in this study exhibited many similarities with the type specimen. For example, it also infects a very similar species of cockroach, kills its hosts on the underside of leaves, attached by fungal structures, and the ascomata emerge laterally from the host’s thorax. Furthermore, the ascomata are very similar in macro-morphological features. Unfortunately, we could not assess the micro-morphological features in this study and thus, to avoid any ambiguities, we are calling our material *O.* cf. *blattae*. We made efforts to sequence the original material from 1924, but all our attempts failed. As this is the type species of the genus *Ophiocordyceps*, further efforts are needed to fix the phylogenetic placement of the type species and thus, re-discuss the systematics of the whole genus. We have considered to propose the specimen used in this study as the epitype of *O. blattae*, however, since it was collected in Thailand, not Sri Lanka as it was originally found by Petch, future efforts might address this issue. However, herein we provide a good perspective for future efforts and revealed another clade likely bearing cryptic species within *Ophiocordyceps* on cockroaches.

## CONCLUSION

Japan may harbour one of the richest reservoirs of entomopathogenic fungi in the world. In no other country is there an amateur society devoted to collecting and illustrating them. We still know very little about these organisms and their ecological roles in the environment and dynamic associations with host insects, including blattodean-associated ones such as *O. salganeicola*. Therefore, it is crucial to understand their true diversity with the invaluable help by such amateur and professional mycologists. As we move forward and describe more and more species through microscopic and molecular tools, new insights into the evolutionary origins of these organisms are being revealed, as well as their ecological associations with the insect hosts. Currently, there is still a substantial gap in our knowledge about insect ecology and fungal biology within the context of host-parasite interactions and their life-cycles. In this study, our goal was to describe an ecologically rare fungal species parasitizing unique social insects, and to provide some insights into their evolution by considering natural histories of both the parasite and its host. Thereby, our study contributes to the understanding of one of the most prolific and diverse groups of entomopathogenic fungi, the genus *Ophiocordyceps*, and incidentally shed new light on the origins of the economically important *O. sinensis*.

## Data Availability

The dataset analyzed in this study is available from the corresponding author upon request.

## Abbreviations

CTAB: Cetyl Trimethyl Ammonium Bromide
Min: Minute
Sec: Seconds
ml: Milliliter
mm: Millimeter
RAxML: Randomized Axelerated Maximum Likelihood
rpm: Rotations per minute
μl: Microliter
